# LGBT OLDER ADULTS AND THEIR PETS IN SOUTHERN APPALACHIA: RESULTS OF QUALITATIVE RESEARCH AND FUTURE RESEARCH DIRECTIONS

**DOI:** 10.1093/geroni/igz038.729

**Published:** 2019-11-08

**Authors:** Maureen MacNamara

**Affiliations:** Appalachian State University, Boone, North Carolina, United States

## Abstract

This session reports on older LGBT adults’ perspectives of roles that pets played in relation to older adults’ aging-in-place experiences. The goal of this study is to advance understanding of the role of pets in the lives of older LGBT adults living in rural communities. The qualitative study used individual, semi-structured interviews with 11 older adults residing in rural Appalachia. Individuals were recruited using purposive sampling techniques, including recruitment through LGBT friendly service providers, a lesbian listserv, and snowball sampling. The majority of respondents reported that pets played a role in social support and some in social capital. One respondent described a utilitarian relationship with animals that is important to understanding the roles animals may play in rural communities in terms of "belongingness". After reviewing study findings, we will provide recommendations for including information about pets in formal social service delivery systems and conclude with implications for future research.

